# Peptide: MHC-based DNA vaccination strategy to activate natural killer cells by targeting killer cell immunoglobulin-like receptors

**DOI:** 10.1136/jitc-2020-001912

**Published:** 2021-05-20

**Authors:** Pauline Rettman, Matthew D Blunt, Rebecca J Fulton, Andres F Vallejo, Leidy Y Bastidas-Legarda, Laura España-Serrano, Marta E Polak, Aymen Al-Shamkhani, Christelle Retiere, Salim I Khakoo

**Affiliations:** 1 School of Clinical and Experimental Sciences, Faculty of Medicine, University of Southampton, Southampton, UK; 2 Antibody and Vaccine Group, Centre for Cancer Immunology, Faculty of Medicine, University of Southampton, Southampton, UK; 3 Etablissement Français du Sang, Centre-Val de Loire, France

**Keywords:** killer cells, natural, immunogenicity, vaccine, immunity, innate

## Abstract

**Background:**

Natural killer (NK) cells are increasingly being recognized as agents for cancer immunotherapy. The killer cell immunoglobulin-like receptors (KIRs) are expressed by NK cells and are immunogenetic determinants of the outcome of cancer. In particular, KIR2DS2 is associated with protective responses to several cancers and also direct recognition of cancer targets in vitro. Due to the high homology between activating and inhibitory KIR genes to date, it has been challenging to target individual KIR for therapeutic benefit.

**Methods:**

A novel KIR2DS2-targeting therapeutic peptide:MHC DNA vaccine was designed and used to immunize mice transgenic for KIR genes (KIR-Tg). NK cells were isolated from the livers and spleens of vaccinated mice and then analyzed for activation by flow cytometry, RNA profiling and cytotoxicity assays. In vivo assays of NK cell function using a syngeneic cancer model (B16 melanoma) and an adoptive transfer model for human hepatocellular carcinoma (Huh7) were performed.

**Results:**

Injecting KIR-Tg mice with the vaccine construct activated NK cells in both liver and spleens of mice, with preferential activation of KIR2DS2-positive NK cells. KIR-specific activation was most marked on the CD11b+CD27+ mature subset of NK cells. RNA profiling indicated that the DNA vaccine upregulated genes associated with cellular metabolism and downregulated genes related to histone H3 methylation, which are associated with immune cell maturation and NK cell function. Vaccination led to canonical and cross-reactive peptide:MHC-specific NK cell responses. In vivo, DNA vaccination led to enhanced antitumor responses against B16F10 melanoma cells and also enhanced responses against a tumor model expressing the KIR2DS2 ligand HLA-C*0102.

**Conclusion:**

We show the feasibility of a peptide-based KIR-targeting vaccine strategy to activate NK cells and hence generate functional antitumor responses. This approach does not require detailed knowledge of the tumor peptidomes nor HLA matching with the patient. It therefore offers a novel opportunity for targeting NK cells for cancer immunotherapy.

## Introduction

The potential of natural killer (NK) cells for treating cancer is becoming increasingly recognized. Current NK cell therapeutics include adoptive transfer of NK cells, chimeric antigen receptor (CAR)-NK cells, cytokine-based activation of NK cells and checkpoint inhibitors, with methods to specifically target endogenously expressed activating receptors being less common.^1^ In order to develop new methods to target NK cells requires a detailed understanding of the receptor:ligand interactions between NK cells and their cancer targets.^2 3^ One important family of NK cell receptors is the killer cell immunoglobulin-like receptors (KIRs). These form a polymorphic gene family of receptors with MHC class I ligands.^4^ KIRs have been implicated in susceptibility to and the outcome of many different cancers.^5–11^ Thus, targeting the KIR could form part of a therapeutic strategy to treat cancer.^12^

The KIR can be inhibitory or activating. The distinction between these two types is based on their signaling capabilities. In general, activating KIRs have short intracytoplasmic tails and a charged amino acid residue in their transmembrane domain, which recruits the adaptor molecule DAP12, leading to phosphorylation of its immunoreceptor tyrosine-based activation motifs, recruitment of src family members and hence cellular activation.^13^ Conversely, inhibitory KIRs have long intracytoplasmic tails containing immunoreceptor tyrosine-based inhibitory motifs, which can be phosphorylated, recruit SHP1 and this leads to inhibition of cellular activation.^14^

Both activating and inhibitory KIR recognize MHC class I. Thus, MHC class I expression on tumor cells can inhibit killing by NK cells expressing inhibitory KIR, an interaction that has been targeted using the monoclonal antibody lirilumab.^15^ Conversely, tumors expressing MHC class I could theoretically be targeted by NK cells expressing activating KIR, however, to date no tumor-specific ligands have been identified. This is in part because the ligand specificities of the activating KIR have been hard to identify.^16^ Recent work has shown that activating KIR can have an HLA class I-restricted peptide specificity.^17–20^ While T cell receptors have a tight restriction on the peptide:MHC complexes that they bind, the KIRs recognize families of peptide:MHC complexes in a motif-based manner, allowing recognition of multiple peptides and HLA class I allotypes.^21 22^ Utilizing this cross-reactive specificity of KIR offers novel opportunities for immunotherapy in which precise peptide:HLA matching to the target is not required.

KIR2DS2 is an activating receptor that recognizes group 1 HLA-C molecules in combination with different viral and synthetic peptides, and we have recently shown that KIR2DS2 recognizes highly conserved flaviviruses and hepatitis C peptides with an alanine-threonine sequence at the C-terminal −1 and −2 positions of the peptide in the context of HLA-C.^18 23^ Activating KIR has been associated with protective responses against cancer. Specifically, KIR haplotypes containing activating KIR confer protection against relapse of acute myeloid leukemia following bone marrow transplantation, and this has been mapped to the region of the KIR locus that contains KIR2DS2.^24–26^ In cord blood transplantation, the benefit of KIR2DS2 is augmented if the recipient of the transplant possess group 1 HLA-C allotypes, the putative ligands for KIR2DS2.^27^ KIR2DS2 has also been associated with protection against a number of solid tumors including cervical neoplasia, breast cancer, lung cancer, colorectal cancer and hepatocellular carcinoma.^6 7 28–30^ In vitro, recognition of cancer cell lines for KIR2DS2 has been observed, but is not specific and also encompasses inhibitory KIR2DL2/3.^31^ Additionally, KIR2DS2-positive NK cells appear to express higher levels of FcγRIII (CD16), have enhanced functionality and confer enhanced protection against glioblastoma in a xenograft model.^32^ Consistent with this enhanced functionality, in a clinical trial of an anti-GD2 antibody in neuroblastoma, KIR2DS2-positive patients had improved survival compared with KIR2DS2-negative patients.^33^

Targeting KIR2DS2 in an immunotherapeutic strategy is challenging as it shares more than 98% sequence homology with the inhibitory receptors KIR2DL2 and KIR2DL3, making antibody-based therapeutics challenging. However, KIR2DS2 does have a distinct peptide:MHC specificity, suggesting that there is potential for developing peptide-based approaches to activate NK cells in a manner analogous to targeting cytotoxic T cells.^34^ Furthermore, as it has a broad peptide:MHC class I specificity, it has the potential to recognize multiple different peptide:MHC combinations, consistent with observations of protection in both viral diseases and cancer.^18 27^ To investigate the therapeutic potential of targeting activating KIR in a peptide-dependent manner, we have tested a DNA vaccine strategy to activate NK cells through KIR2DS2.

## Methods

### Mice vaccination and tumour models

KIR transgenic mouse expressing a complete human KIR B haplotype on a C57BL6 background MHC class I–deficient Kb^−/−^ Db^−/−^ were a kind gift from J Van Bergen and kept under specific pathogen-free conditions.^35^ DNA constructs were made expressing HLA-C*0102 alone or linked with the T2A self-cleaving peptide and peptide and cloned into pIB2.^18^ For the B16 model, mice were injected intramuscularly with 50 µg DNA; 2.5×10^5^ B16F10 cells into the mice left flank, and 50 µg of DNA plasmid on days 0 and 7. For the Huh7 model, NSG mice were injected with 2×10^6^ Huh7-C*0102 cells subcutaneously and then 1×10^6^ purified NK cells from 2 weeks vaccinated KIR transgenic (KIR-Tg) mice spleen were injected intravenously on days 0 and 14. NK cells were purified using MACS technology (NK Cell Isolation Kit II, Miltenyi Biotec). Purity was > 90% NK cells with <3% CD3+ T cells.

### Histology

Formalin-fixed paraffin-embedded sections were generated from muscle tissue isolated 1 week after the second vaccination. Five micrometer sections were cut, dewaxed and incubated with 0.5% hydrogen peroxidase to block endogenous peroxidase activity. Following blocking with avidin-biotin blocking solution (Vector Labs) and then with 1% bovine serum albumin 20% fetal calf serum in Dulbecco’s modified Eagle’s medium (DMEM), sections were stained with 1:100 anti-HLA class I antibody (Invitrogen), followed by 1:800 biotinylated goat anti-rabbit secondary antibody (Vector Labs) and then avidin-biotin peroxidase (Vector Labs). Slides were developed with DAB substrate (BioGenex) then counterstained with hematoxylin blue for 30 s.

### Cell lines

HLA class I‐deficient 721.221 lymphoblastoid EBV‐B cells were cultured in R10 medium (RPMI 1640 supplemented with 1% penicillin-streptomycin (Life Technologies) and 10% heat inactivated fetal bovine serum (FBS; Sigma)); 721.221 cells were transduced with the pIB2 constructs to express HLA-C*0102 alone or together with peptides LNPSVAATL, IVDLMCHATF. B16F10 cells were cultured in DMEM with 1% penicillin-streptomycin (Life Technologies) and 10% FBS.

### Flow cytometry analyses

Murine lymphocytes were stained using anti-mouse CD3ε-PE, NK1.1-BV421, CD11b-APC-Cy5, CD27-BV510, KLRG1-PECy7 (Biolegend). The 1F12-FITC antibody was used to selectively stain KIR2DS2.^36^ For CD107a assays, freshly isolated splenocytes were cultured with anti-CD107a AF647 (Biolegend) and GolgiStop (BD Biosciences) prior to staining. Cells were stained with 1F12-FITC, CD3-PE, NK1.1-BV421 (Biolegend). For the IFN‐γ secretion assay, splenocytes were surface stained with 1F12-FITC, CD3-PE, NK1.1-BV421 antibodies, fixed and permeabilized using BD Cytofix/Cytoperm buffers and then stained with anti-mouse IFNγ-APC (Biolegend). Events were acquired on Aria II (BD Biosciences) using the FACSDiva software (BD Biosciences) and analyzed with FlowJo software (Treestar). For Huh7-HLA-C*0102 cytotoxicity assays, target cells were co-incubated with the indicated NK cell population for 4 hours. Cells were then stained with LIVE/DEAD stain (ThermoFisher Scientific) and analyzed by flow cytometry, gating on the target cell population identifiable by GFP within the HLA-C*0102 construct.

### RNA Seq data analysis and processing

RNA was isolated from murine splenic NK cells using the RNeasy Kit (QIAGEN) and prepared the QIAseq UPX 3’ Transcriptome Kit (QIAGEN); 10 ng purified RNA was used for the next generation sequencing (NGS) libraries. The library pool was sequenced on a NextSeq500 instrument. Raw data was de-multiplexed and FASTQ files generated using bcl2fastq software (Illumina). FASTQ data were checked using the FastQC tool (https://www.bioinformatics.babraham.ac.uk/projects/fastqc/). For differential gene expression analysis, raw counts from RNA-Seq were processed in Bioconductor package EdgeR,^37^ variance was estimated and size factor normalized using TMM. Genes with a minimum of four reads at minimum of 40% samples were included in the downstream analyses. Differentially expressed genes (DEGs) were identified applying significance threshold FDR p<0.05. Blind, normalized log2 values calculated by EdgeR were used for PCA and to calculate Euclidean distances for hierarchical clustering using Ward’s method. For heatmaps, the normalized log2 values of all high-fold change peaks were used to hierarchically cluster peak regions into seven clusters, with the top 100 most variable genes (based on calculated variance across all samples). Gene ontology and pathway enrichment analysis were done using CAMERA,^38^ Ensemble of Gene Set Enrichment Analyses (EGSEA)^39^ and ToppGene.^40^ All analyses used default settings considering mouse orthologues from the MSigDB V.5.2 databases retrieved from http://bioinf.wehi.edu.au/software/MSigDB/. Only pathway terms with a minimum of 25 genes were considered and used for multiple hypothesis correction. Enriched pathways were filtered for those that showed p_adj_<0.05 for both p values as calculated for the construct C*0102-IVDL versus C*0102-AAA in KIR2DS+ and KIR2DS2− NK cell populations. Pathway results were further filtered on those that showed the lowest p_adj_ values.

### Immunoblotting

NK cells were obtained from murine spleens using an NK Cell Isolation Kit (Miltenyi Biotech) and lysed in NP40 Cell Lysis Buffer (Fisher Scientific UK). Primary antibodies from Cell Signaling Technology against Di-Methyl-Histone H3 (Lys4) (#9725), Tri-Methyl-Histone H3 (Lys27) (#9733), Histone H3 (#14269) and actin (#3700) were used in conjunction with HRP-conjugated secondary antibodies. Proteins were visualized using ChemiDoc-It Imaging system and VisionWorks software.

### Statistical analysis

Experimental statistical analyses were performed using GraphPad Prism V.7.0 software. Student’s two-tailed t-test was used for comparison between two groups and two-way analysis of variance with post hoc analysis were used to compare more than two groups. Data were considered statistically significant at p<0.05. For the tumor model, statistical comparisons between survival to the humane end point were performed by log-rank test (Mantel-cox).

## Results

### Activation of NK cells using a peptide:MHC DNA vaccine to target KIR2DS2

**Figure 1:**
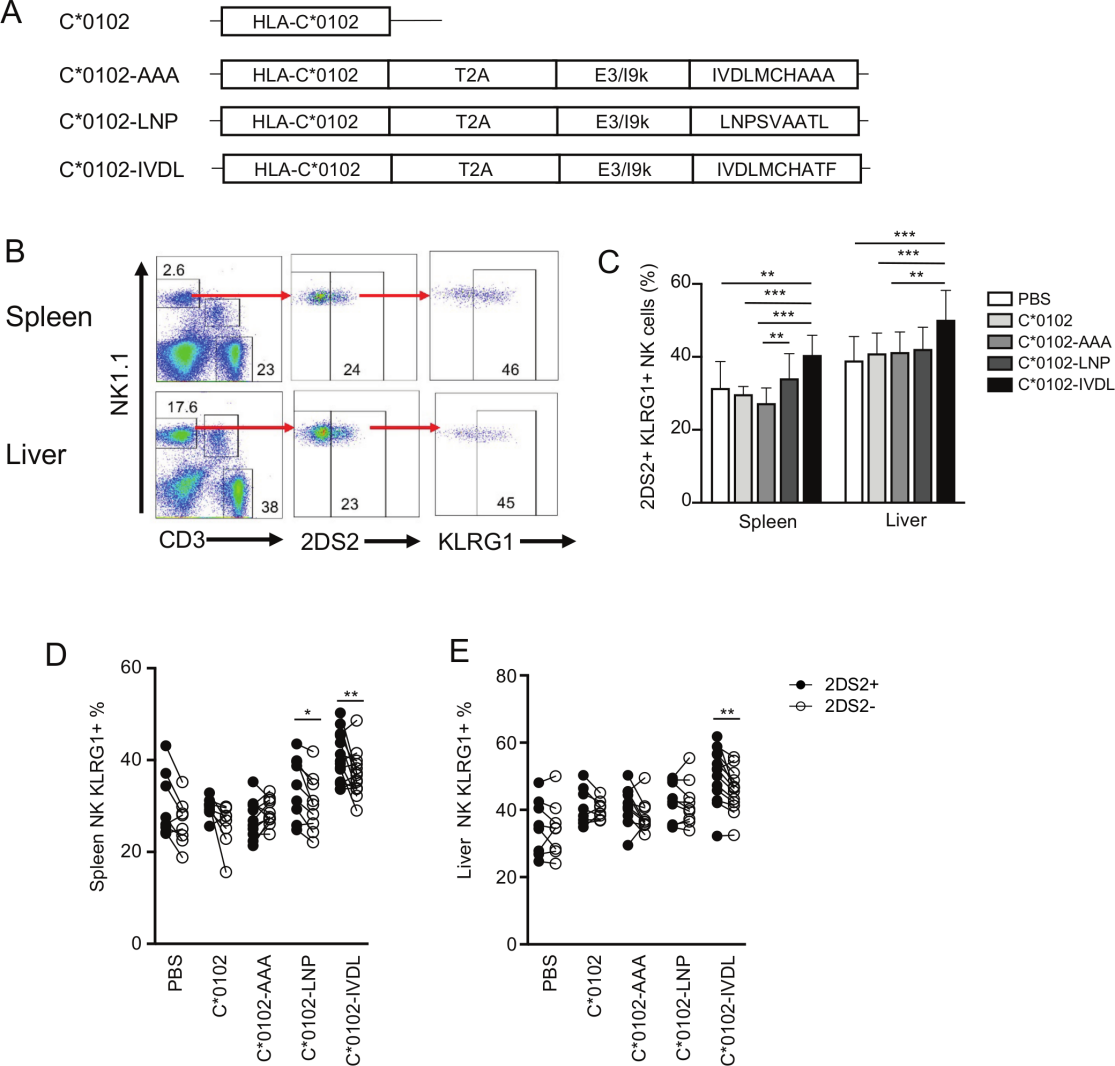
A peptide:MHC DNA vaccine that targets KIR2DS2 activates NK cells. (A) The conformation of the constructs used in this study to inoculate the mice. (B) Gating strategy for KLRG1 on KIR2DS2+ NK cells derived from the spleens (top panels) and livers (lower panels) of KIR-Tg mice. KIR2DS2+ NK cells were identified using the antibody 1F12 and the numbers indicate the percentage positive cells in the gate. (C) The frequency of KLRG1 expression on KIR2DS2+ NK cells in the spleen and livers of KIR-Tg mice vaccinated with DNA plasmids containing HLA-C*0102 (C*0102, light gray bars), HLA-C*0102 plus IVDLMCHATAAA (C*0102-AAA, gray bars), HLA-C*0102-LNPSVAATL (C*0102-LNP, dark gray bars), HLA-C*0102-IVDLMCHATF (C*0102-IVDL, black bars) and compared with PBS alone control mice (white bars). (D, E) Comparison of KLRG1 frequencies on KIR2DS2+ (filled circles) or KIR2DS2− (open circles) CD3-NK1.1+NK cells in the spleens (D) and livers (E) following vaccination. All analyses were performed 1 week following the second vaccination. Comparisons between two groups were made by paired t-test (two groups) (D and E) and two‐way analysis of variance with Dunnett’s test for multiple comparisons to compare individual groups (1C) (*p<0.05, **p<0.01, ****p<0.001). KIR, killer cell immunoglobulin-like receptor; NK, natural killer.

We developed a DNA construct that expresses the peptide:MHC (pMHC) ligand for KIR2DS2 as a single open reading frame (ORF). The rationale for this was that KIR2DS2 is a cross-reactive receptor that recognizes different pMHC ligands and that in order to target KIR2DS2 for therapeutic benefit it is therefore not required to know the peptide ligand of the tumor being targeted or to match for the HLA of the patient. In our previous work, we developed a method for expressing an MHC complex together with a cognate peptide as a single ORF.^18^ These constructs contained HLA-C*0102 linked to a viral peptide and separated by a T2A self-cleaving sequence^41^ and with an optimal E3/19K endoplasmic reticulum (ER) targeting sequence upstream of the peptide.^42^ No modifications to these published sequences were made, and these constructs were shown to successfully present peptides to KIR2DS2-positive NK cells.^23^ The constructs were made with two previously described KIR2DS2-binding peptides: LNPSVAATL (C*0102-LNP) and IVDLMCHATF (C*0102-IVDL) and cloned in to the pIB2 expression vector (figure 1A). The DNA was dissolved in PBS and used to immunize KIR-Tg mice.^35^ No additional vehicles or adjuvants were used. These mice express a human KIR haplotype B locus that includes KIR2DS2 and are backcrossed onto an MHC class I negative background. An MHC class I-deficient mouse was used to avoid presentation of peptides onto endogenous murine MHC class I and hence confounding T cell or NK cell responses. In these animals, KIR2DS2 expression is unaffected by the absence of an endogenous HLA-C ligand.^35^ KIR-Tg mice were injected intramuscularly with 50 µg of DNA and expression of MHC class I analyzed by immune histochemistry 1 week after the final injection. This demonstrated expression of the construct within muscle tissues (online supplemental figure S1). KIR-Tg mice were then injected intramuscularly with 50 µg of DNA, without additional adjuvant, weekly for 2 weeks. Mice were sacrificed 1 week after the final injection, and splenocytes and hepatic lymphocytes isolated using KLRG1 expression as a marker of NK cell activation that is also associated with NK cell proliferation and maturation.^43 44^ Results were compared with DNA constructs encoding HLA-C*0102 alone or DNA encoding HLA-C*0102 in combination with a control peptide, IVDLMCHAAA (C*0102-AAA), in which the P9 and P10 residues of the peptide IVDLMCHATF had been mutated to alanine. HLA-C*0102 has a preference for peptides with a carboxy terminal residue of leucine and these substitutions are predicted to reduce binding to HLA-C as defined by analysis by netMHCpan V.4.1 (http://www.cbs.dtu.dk/services /NetMHCpan/), reducing the rank binding to HLA-C*0102 from 8% for IVDLMCHATF to 33% for IVDLMCHAAA. As our previous work has shown that binding of KIR2DS2 to HLA-C*0102 requires a peptide with threonine at the carboxy terminal −1 position, together these substitutions are predicted to abrogate binding to KIR2DS2 and HLA-C*0102. Injection with DNA encoding viral peptides induced activation of splenic NK cells as indicated by KLRG1 expression, but was greatest in the C*0102-IVDL group (40%) compared with C*0102-AAA (28%), p<0.001 (figure 1B, C). While this effect is modest, it was robust and represents activation of ~10% of NK cells in these animals. However, we did not observe a specific increase in the frequency of KIR2DS2-positive NK cells (data not shown).

10.1136/jitc-2020-001912.supp1 Supplementary data

In paired analyses, KLRG1 expression was upregulated to a significantly greater extent on KIR2DS2-positive (KIR2DS2+) versus KIR2DS2-negative (KIR2DS2−) splenic NK cells (p<0.01 for C*0102-IVDL and p<0.05 for C*0102-LNP), but not by control constructs (figure 1D). Activation was more marked in mice injected with C*0102-IVDL compared with C*0102-LNP, consistent with the stronger binding in vitro noted previously in tetramer binding experiments.^18^ Furthermore, activation of hepatic NK cells was noted only in experiments using the C*0102-IVDL construct, with the expression of KLRG1 being significantly higher on the KIR2DS2+ versus KIR2DS2− population (p<0.01) (figure 1E).

### Peptide:MHC vaccination upregulates KLRG1 expression on KIR2DS2-positive mature splenic NK cells

**Figure 2:**
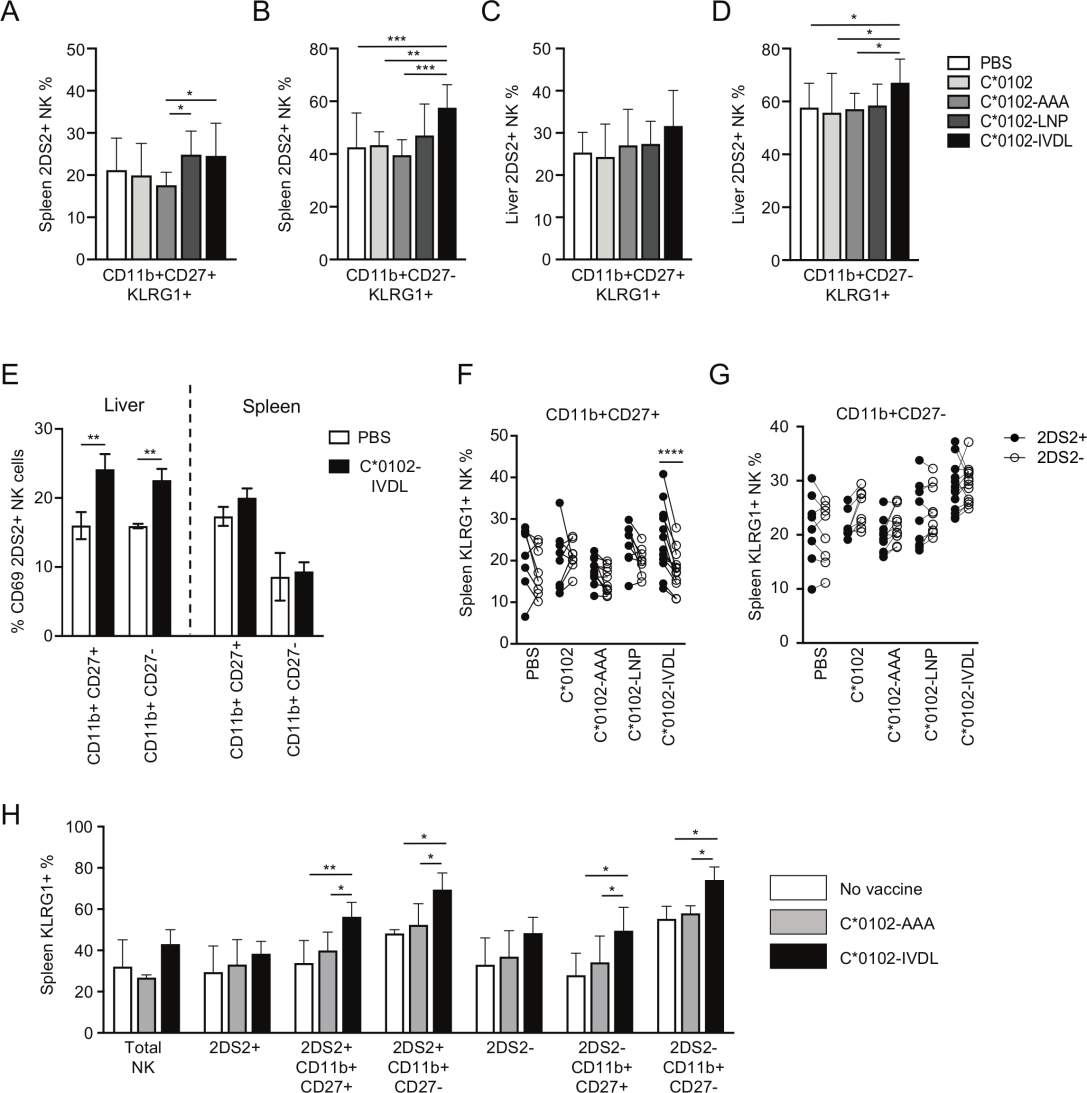
Vaccination activates mature and terminally differentiated NK cells. KIR-Tg mice were injected with two doses of the indicated DNA construct 1 week apart and then assessed for expression of KLRG1 on CD11b, CD27 NK cell subsets from the spleens and livers 1 week after the final injection. (A–D) KLRG1 on splenic CD11b+CD27+ (A) and CD11b+CD27− (B) NK cells and on hepatic CD11b+CD27+ (C) and CD11b+CD27− (D) NK cells following vaccination. N=7–14 mice per group. (E) CD69 expression on KIR2DS2-positive CD11b CD27 NK cell subsets (n=3 per group). (F, G) Comparison of KLRG1 expression on KIR2DS2+ and KIR2DS2− splenic NK cells in the CD11b+CD27+ (F) and CD11b+CD27− (G) subpopulations. N=7–14 mice per group. (H) KIR-Tg mice crossed with C57BL/6 mice were injected with two doses of the indicated DNA construct 1 week apart and then assessed for expression of KLRG1 on total NK cells, and CD11b, CD27, NK cell 2DS2+ and 2DS2− subsets from their spleens. N=4–6 mice per group. Comparisons between two groups were made by paired t-test (two groups) (E–G) and two‐way analysis of variance with Dunnett’s test for multiple comparisons to compare individual groups (A–D and H) (*p<0.05, **p<0.01, ***p<0.005, ****p<0.001). KIR, killer cell immunoglobulin-like receptor; NK, natural killer.

Maturation of NK cells can be characterized by expression of CD11b and CD27, with CD11b−CD27+ NK cells being classified as the least mature, CD11b+CD27+ NK cells as mature and CD11b+CD27− NK cells terminally differentiated. Overall, there were no substantial changes in the relative proportions of the different CD11b/CD27 subsets induced by these constructs (online supplemental figure S2). KLRG1 was upregulated by C*0102-IVDL vaccination on CD11b+CD27+ splenic 2DS2+NK cells compared with C*0102-AAA: 27% vs 18% (p<0.05), respectively, and on terminally mature CD11b+CD27− splenic 2DS2+ NK cells compared with both C*0102-AAA and C*0102: 62% vs 40% (p<0.001) and 46% (p<0.005), respectively (figure 2A, B). However, effects on hepatic lymphocytes were less clear, with upregulation of KLRG1 only noted on terminally differentiated CD11b+CD27− NK cells in the C*0102-IVDL group only (p<0.05 vs control vaccinations) (figure 2C, D). Conversely, we observed upregulation of CD69 on hepatic, but not splenic CD11b+CD27+ and CD11b+CD27− 2DS2+ NK cells (figure 2E). This difference may reflect the kinetics of CD69 versus KLRG1 expression and also the observation that CD69 can mark tissue-resident NK cells, in addition to being a marker of activation.

Specific activation of KIR2DS2+ versus KIR2DS2− NK cells was most marked on the CD11b+CD27+ splenic NK cells (p<0.001) compared with terminally differentiated CD11b+CD27− NK cells (figure 2F, G). Analysis of draining lymph nodes demonstrated a trend toward an increase in KIR2DS2-positive NK cells and also toward expression of KLRG1 on NK cells in the C*0102-IVDL group compared with the C*0102-AAA group (online supplemental figure S3). After 4 weekly injections, we observed activation of NK cells, with upregulation of KLRG1 on splenic and hepatic NK cells with both peptide-containing constructs and KIR2DS2-specific activation in the CD11b+CD27+ double-positive population with C*0102-IVDL (online supplemental figure S4). However, at this timepoint there was no significant difference between the C*0102-LNP and C*0102-IVDL constructs. This lack of difference may reflect that a ceiling for activation using this strategy may have been reached or that after four doses more apoptosis is induced by the C*0102-IVDL than the C*0102-LNP construct.

**Figure 3:**
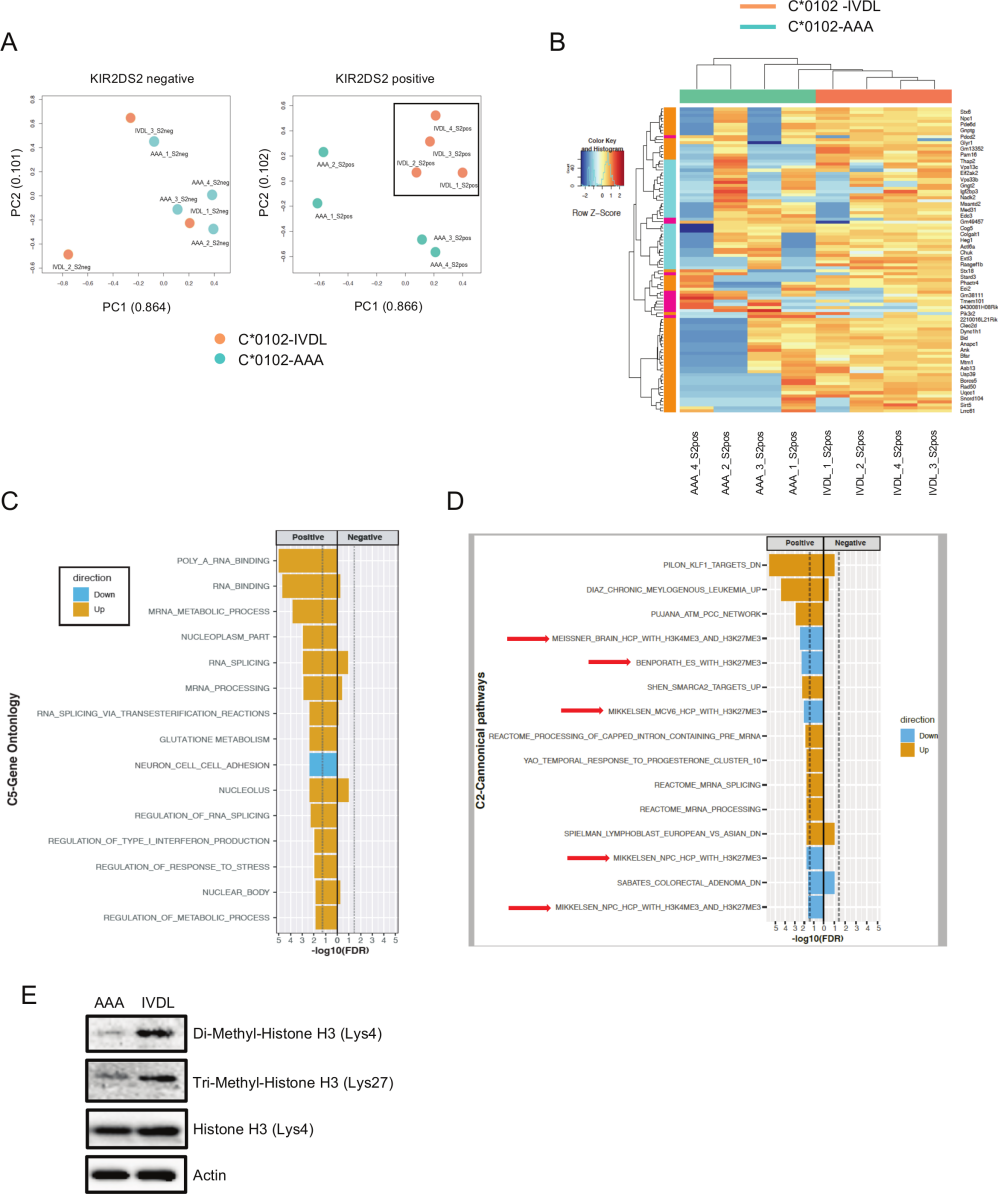
Transcriptomic analysis of NK cells following DNA vaccination. (A) Principal component analysis (PCA) of whole NK cell transcriptomes from C*0102-IVDL and C*0102-AAA vaccinated mice taken 1 week after the second vaccination. KIR2DS2-negative NK cells from both groups are shown in the left panel and KIR2DS2-positive NK cells in the right panel. Counts were normalized and filtered using EdgeR. The first two components of the PCA are shown. (B) Heatmap of the top 100 differentially expressed genes derived from the comparison of KIR2DS2-positive NK cells from C*0102-IVDL and C*0102-AAA vaccinated mice. (C, D) EGSEA analysis of C5 gene ontology (C) and C2 canonical pathway (D) signatures comparing KIR2DS2+ and KIR2DS2− NK cell populations in both C*0102-IVDL and control-vaccinated mice. Effect significances were calculated individually for each arm of the study and the plots indicate the overall effects of vaccination on KIR2DS2+ NK cells in the C*0102-IVDL vaccinated mice (‘positive’) compared with the other three groups (‘negative’). The color denotes the direction of the change and the size of the bar represents the -Log10(FDR). All categories shown were significant at FDR<0.05. (E) Western blot analysis of histone 3 marks (H3K4me2 and H3K27me3) on purified NK cells from the spleens of vaccinated mice 1 week after the second vaccination with either C*0102-AAA (AAA) or C*0102-IVDL (IVDL). NK, natural killer.

Education of NK cells in the mouse is determined by interactions between the Ly49 inhibitory receptors and their MHC class I ligands. Therefore, to confirm that activation of NK cells could be induced on educated NK cells, we crossed the KIR-Tg mice with wild-type C57BL/6 mice to provide MHC ligands for the murine Ly49 receptors and immunized with two doses of DNA. Consistent with findings in the MHC-deficient mouse, we observed KLRG1 upregulation on mature CD11b+CD27+ KIR2DS2+ and terminally differentiated CD11b+CD27− KIR2DS2+ NK cells (p<0.01 and p<0.05, respectively) (figure 2H).

NK cells from C*0102-IVDL and C*0102-AAA vaccinated mice were profiled by RNA-Seq 1 week following vaccination (see online supplemental figure S5 for gating strategy). Principal component analysis (PCA) showed that KIR2DS2+ NK cells from C*0102-IVDL-vaccinated mice formed a discrete cluster to KIR2DS2+ NK cells from C*0102-AAA vaccinated mice in contrast to KIR2DS2− NK cells (figure 3A). Those mice receiving C*0102-IVDL had upregulation of genes in pathways associated with cellular metabolism compared with those receiving control C*0102-AAA vaccination (figure 3B). Differential gene expression analysis identified 42 DEGs (FDR<0.05) between the KIR2DS2+ NK cells from the C*0102-IVDL versus control groups (online supplemental table S1). These included upregulation in genes associated with: RNA binding and splicing; metabolism, especially glutathione metabolism; and regulation of IFN alpha (figure 3C). Additionally, we observed downregulation of genes related with histone H3 dimethylation at K4 (H3K4me2) and trimethylation at K27 (H3K27me3), consistent with a change in transcriptional regulation induced by vaccination (figure 3D). Purified NK cells from the spleens of vaccinated mice had upregulation of both H3K4me2 and H3K27me3 in IVDL mice compared with control (figure 3E). Changes in H3K4 and H3K27 methylation have been associated with both NK cell activation and maturation.^45 46^ In particular, promoters with both H3K4 and H3K27 methylation marks are considered to be in a poised, ready to transcribe state, which is consistent with our analysis at 7 days post-vaccination.^47^ While H3K4me2 and H3K4me3 marks are generally concordant in T cell analyses, H3K4me2 marks are associated with genes that are rapidly transcribed after stimulation consistent with such a poised state.^48 49^ Detailed temporal analysis by chromatin immunoprecipitation is required to further clarify the changes in histone methylation induced by vaccination.

10.1136/jitc-2020-001912.supp2 Supplementary data

### A KIR targeting vaccine augments NK cell functions

**Figure 4:**
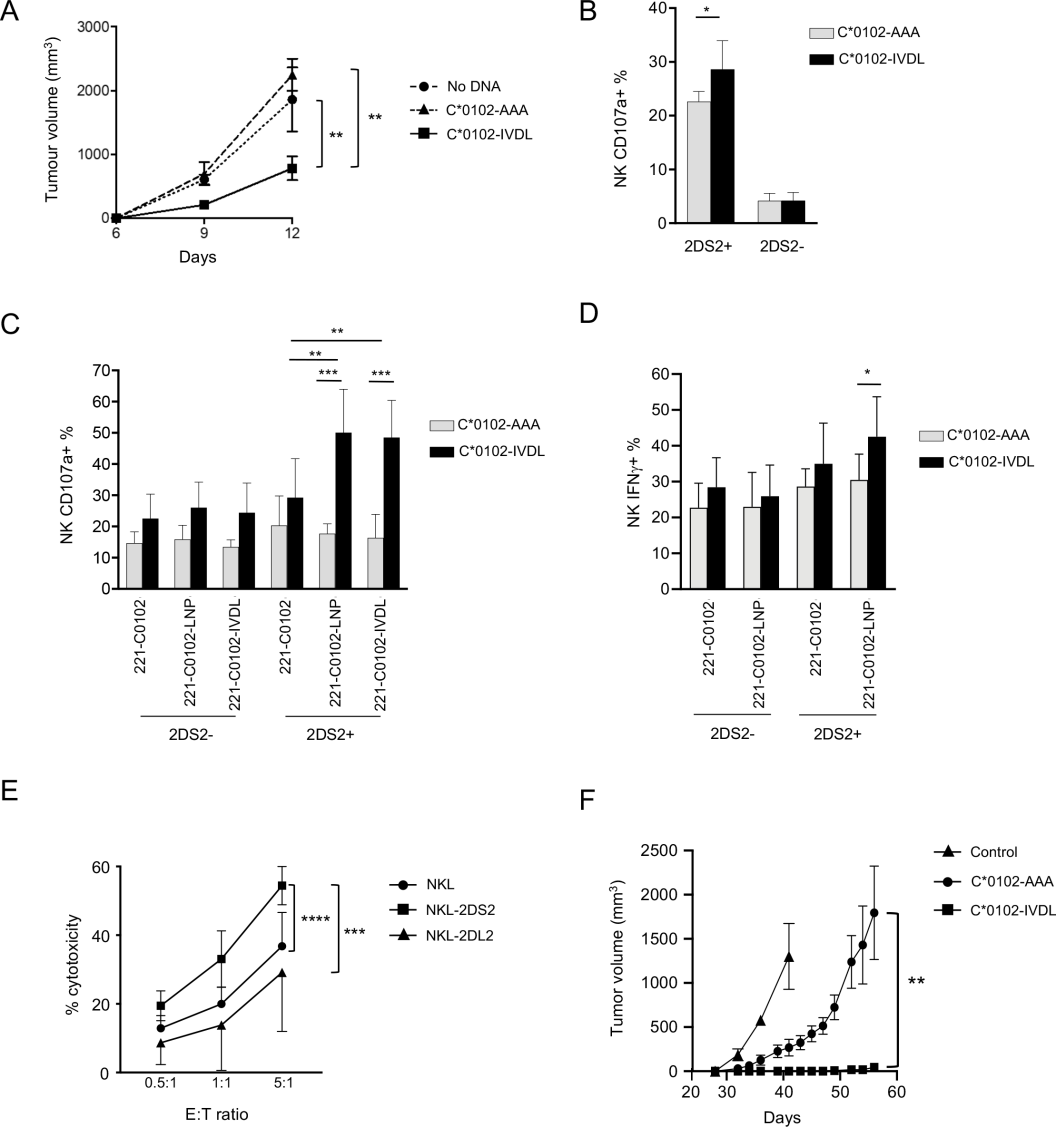
DNA vaccination induces functional NK cell responses. (A) KIR-Tg mice were injected subcutaneously with B16F10 melanoma cells on day 0 and then vaccinated intramuscularly with C*0102-IVDL (squares) or C*0102-AAA (triangles) on days 0 and 7 or untreated (circles) and tumor volume measured (n=4 mice per group: one of two independent experiments). (B, C, D) Mice were injected intramuscularly with C*0102-IVDL (black bars) or C*0102-AAA (gray bars) on days 0 and day 7 and then NK cells purified from the spleens on day 14 for in vitro assays of activation. (B) Degranulation of KIR2DS2+ and KIR2DS2- KIR-Tg NK cells to B16F10 melanoma cells (n=4 mice per group). (C) Degranulation of NK cells from KIR-Tg mice to human 721.221 cells expressing HLA-C*0102 alone (221 C*0102) or HLA-C*0102 in combination with the peptide: LNPSVAATL (221 C*0102-LNP) or IVDLMCHATF (221 C*0102-IVDL) and (D) IFNγ expression following incubation with 221 C*0102 and 221 C*0102-LNP cells (n=7–8 mice per group). (E) Killing of Huh7-C*0102 hepatoma cells by NKL cells either untransfected (NKL) or transfected with KIR2DS2 (NKL-2DS2) or the inhibitory receptor KIR2DL2 (NKL-2DL2) at the indicated effector to target (E:T) ratios. Cytotoxicity was determined by flow cytometry using the LIVE/DEAD stain. Shown are the results of two independent experiments performed in triplicate. (F) NK cells from KIR-Tg mice vaccinated either with C*0102-IVDL or C*0102-AAA as a peptide control were adoptively transferred into NSG mice inoculated subcutaneously with Huh7-C*0102 hepatoma cells and tumor volume was measured (n=4 mice per group, one of two independent experiments). The control mice (n=3) did not receive any NK cells. Comparisons were by Student’s t-test (two groups) (B, D) or two‐way analysis of variance with Dunnett’s test for multiple comparisons to compare individual groups (A, C, E and F). For all plots *p<0.05, **p<0.01, ***p<0.005, ****p<0.0001. KIR, killer cell immunoglobulin-like receptor; NK, natural killer.

To test for functional effects of our DNA vaccination strategy, KIR-Tg mice were inoculated subcutaneously in the flank with B16F10 melanoma cells and injected intramuscularly with DNA on the same day and 1 week later. Growth of B16F10 cells was significantly attenuated by day 12 in mice given C*0102-IVDL compared with those given C*0102-AAA or unvaccinated (p<0.02) (figure 4A). In vitro, KIR2DS2+ NK cells, but not KIR2DS2− NK cells, from C*0102-IVDL-vaccinated mice had increased degranulation to B16F10 cells compared with the control-vaccinated mice (p<0.05) (figure 4B). As B16F10 cells do not express HLA-C, these data indicate that DNA vaccination with C*0102-IVDL activates NK cells and induces MHC class I unrestricted, hence KIR2DS2 independent, responses. This is relevant as many cancers can downregulate MHC class I.

To identify if peptide-specific NK cell responses were generated using this strategy, NK cells from vaccinated mice were tested against the MHC class I-negative target human cell line 721.221 transfected with either HLA-C or a construct of HLA-C in combination with the peptides LNPSVAATL and IVDLMCHATF.^18^ KIR2DS2+ NK cells from mice vaccinated with C*0102-IVDL demonstrated increased activity against 721.221 cells expressing HLA-C*0102 in combination with both KIR2DS2-binding peptides compared with 721.221 cells transfected with HLA-C*0102 alone (p<0.01 for both LNP and IVDL targets) (figure 4C). No effect was observed for KIR2DS2− NK cells. Similarly, we observed an increase in IFNγ secretion for the KIR2DS2+ NK cells from IVDL vaccinated mice when incubated with the C*0102-LNP target compared with KIR2DS2+ NK cells from AAA-vaccinated mice (42.5% vs 30.5%, p<0.05) (figure 4D). Thus, activation using a peptide:MHC strategy can generate both specific and cross-reactive peptide responses.

To identify if this strategy could recognize a human MHC class I target in vivo, we used the Huh7 hepatocellular carcinoma cell line transfected with the KIR2DS2 ligand HLA-C*0102. In vitro killing assays demonstrated that NKL cells transfected with KIR2DS2 killed Huh7-C*0102 cells to a greater extent than untransfected NKL cells (p<0.0001) (figure 4E), thus validating the cell line as KIR2DS2-specific target. As the KIR-Tg mice are not permissive for human tumors, we used an adoptive transfer model to test the effects of our DNA vaccine on Huh7-C*0102 cells. KIR-Tg mice were vaccinated with two doses of the DNA vaccine C*0102-IVDL or the control vaccine weekly, and then purified splenic NK cells containing <1% CD3^+^ T cells, were adoptively transferred into immunodeficient NSG mice, which had been inoculated with Huh7-C*0102 cells. We observed a significantly delayed growth of the tumor in mice that received C*0102-IVDL-stimulated NK cells compared with control vaccine concomitant (figure 4F). Thus, stimulation of NK cells via KIR2DS2 can generate anti-cancer reactivity against HLA-C expressing human tumor cells.

## Discussion

We have developed a novel strategy to activate NK cells through KIR2DS2 in a peptide:MHC-dependent manner using a construct that expresses both the cognate MHC and peptide. This strategy may be relevant for other activating KIR, such as KIR2DS1 and KIR2DS4 which have been convincingly shown to have peptide:MHC specificities.^17 19 50^ Additionally, KIR3DS1, which is associated with protection from HIV, is more controversially thought to have a peptide-dependent HLA-B specificity.^51^ Thus, our work may be relevant for a number of different KIR in addition to KIR2DS2.

KIR2DS2-mediated activation generated a cytotoxic immune response against targets both with and without a cognate KIR2DS2-ligand, and thus utility of this strategy as a therapeutic may not be critically dependent on MHC class I expression by the target. Furthermore, previous work has also shown that KIR2DS2+ NK cells can recognize several different cancer targets in vitro, including cell lines derived from prostate, breast and ovarian carcinomas.^31^ This recognition was beta-2-microglobulin independent, suggesting that there may also be non-peptide:MHC class I ligands for KIR2DS2. Additionally, KIR2DS2+ NK cells appear to have a greater potential to mediate antibody-dependent cellular cytotoxicity (ADCC) in vitro and in vivo.^32 33^ Furthermore, as killing of the B16 melanoma cell line is mediated by the activating NK cell receptor NKp46, KIR2DS2-independent anti-tumor responses are also generated. Therefore, as targeting of KIR2DS2 augments NK cell activity and tumor recognition through receptors other than KIR2DS2, it makes KIR2DS2 an attractive target for cancer immunotherapy both alone and in combination with antibody-based therapeutics.

In our experimental model, KIR2DS2 expression is unaffected by the absence of an endogenous HLA-C ligand as NK cell education is driven by inhibitory, rather than activating, receptors for MHC class I.^35^ This could be further investigated in humanized mouse models, with the caveat that this requires administration of human IL-15 to maintain NK cells, and so will require careful interpretation.^52 53^ Despite the absence of a known ligand for KIR2DS2, we observed activation of both KIR2DS2+ and KIR2DS2− NK cells in vivo. This is not unexpected as a DNA vaccine may generate an inflammatory response, thus activating NK cells, and CpG sequences within the DNA vaccine may activate NK cells either directly or indirectly via dendritic cells. Importantly, NK cells are well known to undergo reciprocal activation with dendritic cells, which could lead to cytokine release and further activation of NK cells in a non-specific manner through release of IL-12.^54^ This is consistent with our observations that while there was activation of both KIR2DS2+ and KIR2DS2− NK cells in vivo, activation was preferential for KIR2DS2+ NK cells. The presence of antigen non-specific responses may be important for generating more effective anti-tumor responses and is consistent with a mode of action of NK cells in which they are activated through one receptor and recognize a target through a different one. For instance, killing of the B16F10 melanoma cell line is mediated by NKp46, and hence not MHC class I restricted.^55^ Importantly, although only approximately 10% of NK cells upregulated KLRG1, this was sufficient to generate enhanced anti-tumor responses in KIR-Tg mice.

Furthermore, we demonstrated peptide cross-reactivity of KIR2DS2, as mice vaccinated with IVDLMCHATF were able to recognize the hepatitis C virus peptide LNPSVAATL, consistent with KIR2DS2 recognizing peptides with an AT motif at the carboxy-terminal −1 and −2 positions. Thus, in order to take our findings to the clinic, it is not necessary to identify the ligand on the cancerous cell or to match for HLA class I as KIR2DS2 may bind other group 1 HLA class I allotypes such as HLA-C*0304.^18 20^ The broad specificity of KIR2DS2 also provides an advantage for peptide-based NK cell therapy over T cell pMHC therapeutics, which require more precise pMHC matching. Additionally, as the strategy provides both the peptide and the MHC class I ligand, then no HLA class I matching is required.

Plasmid DNA vaccination is a novel strategy to activate NK cells. To understand its effects in detail would be interesting in additional studies. In particular, we have noted differences between splenic and hepatic NK cells. Baseline KLRG1 expression appears relatively elevated within the liver, which may be the result of homeostatic proliferation or more ready activation of KIR2DS2-positive NK cells, which has been observed in humans.^32 44^ Additionally, we formally tested only splenic NK cells ex vivo but not hepatic or lymph node NK cells, which could also have anti-cancer effects.

Based on our work, we propose the following mechanism for our vaccination strategy. As there is expression of MHC class I in the muscle tissue, we envisage that circulating NK cells can be activated directly within the tissues. NK cells circulate in a primed state, in comparison with T cells, so uptake by professional antigen-presenting cells may not be required for NK cell activation. While there is local muscle damage caused by both vaccination and potentially direct cytotoxicity against the muscle tissue, the activated NK cells can then circulate to the periphery and generate anti-cancer responses. Potentially, some may develop a ‘memory’ phenotype within the liver. We envisage that this strategy could be readily translated to the clinic as a DNA or RNA therapeutic that activates NK cells in vivo to generate anti-cancer responses or augment other immunotherapeutic strategies, including ADCC. It would however only be relevant for the approximately 50% of individuals who express KIR2DS2 as part of a KIR B haplotype. The strategy could also potentially be adapted to a KIR A haplotype through the peptide-selective activating receptor KIR2DS4. Intratumoral injection alone or in combination with other therapeutics such as oncolytic virus therapy may be another potential therapeutic opportunity. However, the intramuscular approach is less invasive and could be administered in primary care settings. Alternatively, to harness the functional activation advantage of KIR2DS2+ NK cells, cell lines expressing peptide:MHC combinations designed to specifically activate KIR2DS2-positive NK cells could also be used in ex vivo expansion protocols for adoptive therapy. This strategy may offer a more controlled activation of NK cells but would be more cumbersome than an injection therapeutic. Activation of NK cells could lead also to off-target effects and killing of healthy cells, but this does not seem to be the case in vivo. For instance in stem cell transplantation, KIR-ligand mismatching, while having a beneficial effect on recurrence, does not lead to increased toxicity against healthy cells, which lack cognate MHC class I receptors for donor NK cells.^56^ Additionally, CAR-NK cell therapy appears to have less side-effects than CAR-T cell therapy, indicating that NK cell therapy in general is well tolerated.^57^

As NK cells are involved in the early immune response to viral infections, and KIR2DS2 recognizes peptides derived from many different viruses, then targeting KIR2DS2 by DNA vaccination may also form part of an anti-viral therapeutic strategy to reduce infection and transmission in the early stages of infection. In conclusion, our work identifies a novel mechanism for activating NK cells and we propose that this may have potential as a novel therapeutic.

## Data Availability

All data relevant to the study are included in the article or uploaded as supplemental information. Data are available upon reasonable request.
