# Supplementary material for: Peptide: MHC-based DNA vaccination strategy to activate natural killer cells by targeting killer cell immunoglobulin-like receptors
Source: J Immunother Cancer. 2021 May 20;9(5):e001912. doi: 10.1136/jitc-2020-001912 (PMC8141441; doi:10.1136/jitc-2020-001912)

BMJ Publishing Group Limited (BMJ) disclaims all liability and responsibility arising from any reliance placed on this supplemental material which has been supplied by the author(s)

Supplemental Figure 1: Immunohistochemistry staining showing expression of C*0102-IVDL construct in muscle

KIR-Tg mice were injected with two doses of C*0102-IVDL construct (left panel) or PBS (right panel) one week apart and thigh muscle removed 7 days after the second injection. Expression of HLA-C was tested by immunostaining with anti-HLA antibody using an immunoperoxidase technique and counterstained with haematoxylin. Magnification was x100, and a 200mM scale bar is indicated.

C*0102-IVDL **PBS**

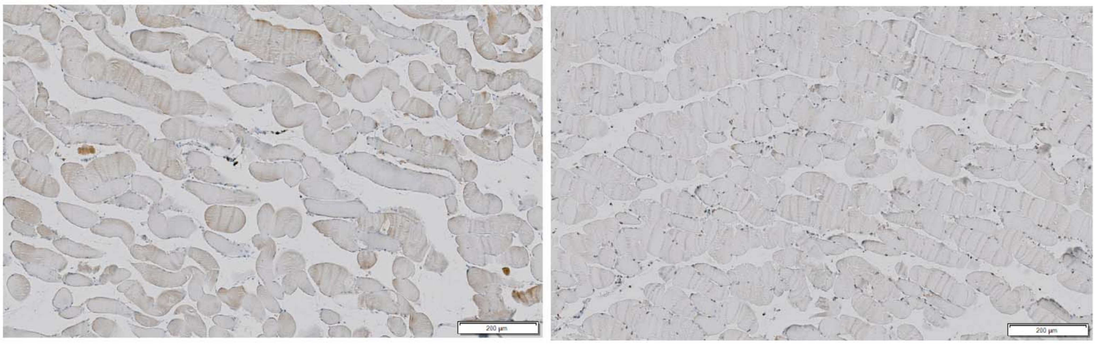

Supplemental Figure 2: CD11b/CD27 subsets from vaccinated mice

KIR-Tg mice were injected with two doses of the indicated DNA construct one week apart and then assessed for maturity as defined by the different CD11b, CD27 NK cell subsets from the spleens one week after the final injection.

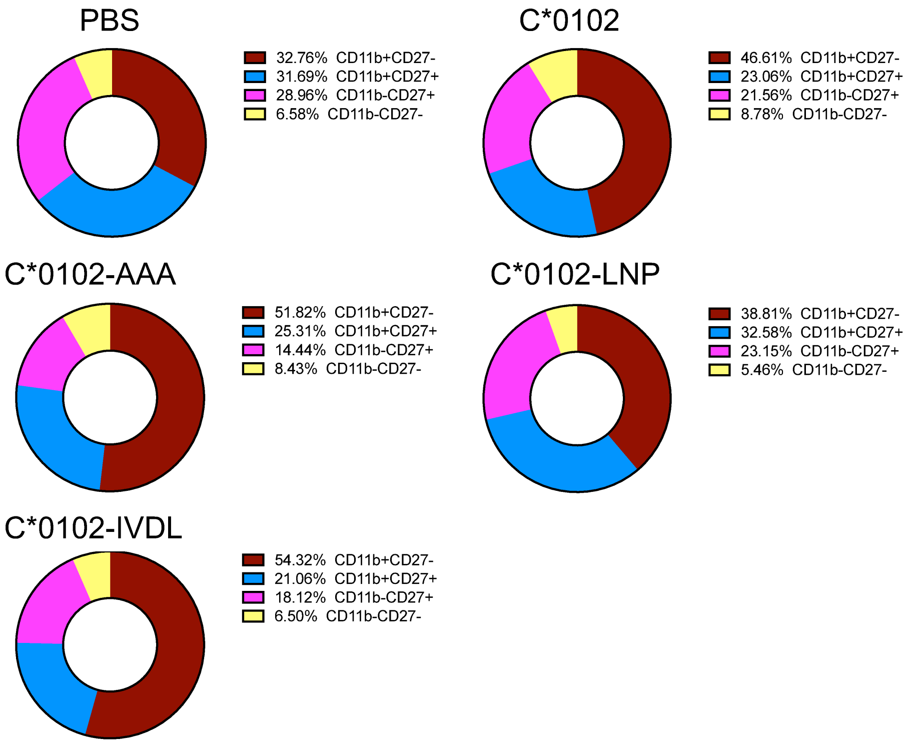

Supplemental Figure 3: Analysis of activation of NK cells in the lymph nodes of KIR-Tg mice

KIR-Tg mice were injected intramuscularly weekly for 2 weeks with DNA constructs encoding HLA-C*0102- IVDLMCHAAA (AAA) or HLA-C*0102-IVDLMCHATF (IVDL). Inguinal lymph nodes were isolated and analysed by flow cytometry. CD3-NK1.1+ NK cells were analysed for KIR2DS2 expression (panel A: n=7 per group) and expression of KLRG1 on KIR2DS2+, or KIR2DS2-, CD11b/CD27 subsets (Panels B and C: 4 mice per group). For all graphs means +/- SEM are shown. There were trends towards increased number of KIR2DS2 –positive cells, and increases in KLRG1 expression in IVDL vaccinated mice, but these did not reach statistical significance.

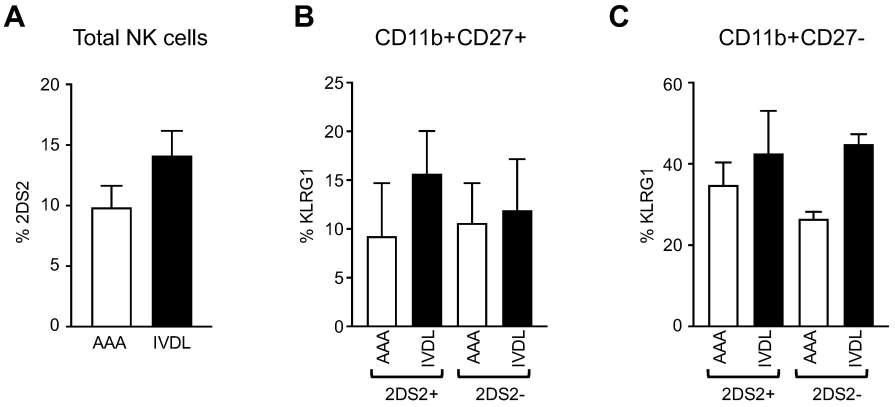

Supplemental Figure 4: Activation of NK cells after DNA vaccination for 4 weeks

KIR-Tg mice were injected intramuscularly weekly for 4 weeks with DNA constructs encoding HLA-C*0102 (white bars), HLA-C*0102-LNPSVAATL (C*0102-LNP; grey bars) or HLA-C*0102-IVDLMCHATF (HLA-C*0102-IVDL; black bars). KLRG1 expression was measured on KIR2DS2+ NK cells and CD11b, CD27 subsets. **A, B**) KLRG1 frequencies on KIR2DS2+ and KIR2DS2- NK cells in spleen **(A)** and livers **(B). C, D).** Frequency of KLRG1 expression on CD11b+CD27+ NK cells in the KIR2DS2+ NK cell subpopulations in the spleens (**C**) and livers **(D)** of vaccinated KIR-Tg mice. **E, F)** Comparison of KLRG1+ expression on CD11b+CD27+ within the KIR2DS2+ and KIR2DS2- NK cells in the spleens (**E**) and livers (**F**) of vaccinated mice. For all experiments n=4 mice per group. A paired t test was used for comparisons between two groups and a 2-way ANOVA with correction used when comparing more than two groups. For all plots *p< 0.05, **p< 0.01, ***p< 0.005.

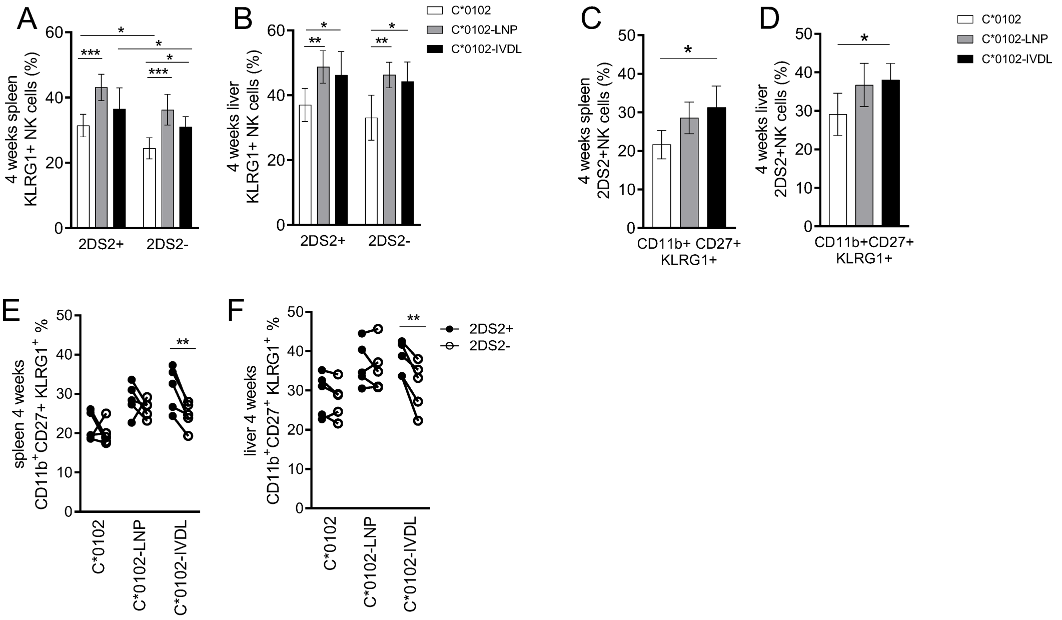

Supplemental Figure 5: Gating strategy for sorting KIR2DS2-positive and -negative NK cells for analysis by RNA seq

Splenocytes from C*0102:IVDL and C*0102:AAA vaccinated mice were isolated and sorted by flow cytometry for analysis by RNAseq. Shown is a flow cytometry plot of the gating strategy used to delineate KIR2DS2+ from KIR2DS2- NK cells. Cells are gated on the CD3-, NK1.1+ sub-population.

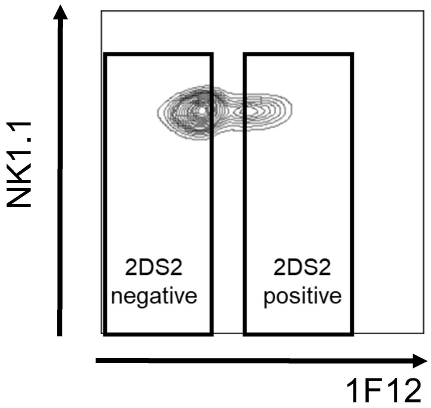

Supplement: Supplementary data [file jitc-2020-001912supp001.pdf]
